# Statin-Induced Autoimmune Myopathy

**DOI:** 10.7759/cureus.13576

**Published:** 2021-02-26

**Authors:** Maryam Nemati, Meena Srai, Rajani Rudrangi

**Affiliations:** 1 Internal Medicine, San Joaquin General Hospital, French Camp, USA; 2 Rheumatology, San Joaquin General Hospital, French Camp, USA

**Keywords:** myopathy, statin-associated autoimmune myopathy, statin

## Abstract

Statins are one of the most widely prescribed drugs in the world. One of the common side effects of statin use is myopathy. We report a case of statin-induced autoimmune myopathy, which is a variant of statin-induced myopathy. A 56-year-old female with a history of hypertension, hyperlipidemia, cerebral aneurysm status post clipping, and seizure disorder presented with progressive muscle weakness. Her initial laboratory results demonstrated an elevated creatine phosphokinase (CPK) of 17,144 IU/L. The patient’s atorvastatin was discontinued and she was placed on high-rate intravenous fluids; however, despite this, her CPK remained elevated. Patient underwent further blood testing for specific autoimmune etiologies. As there was high concern for autoimmune myositis, she was started on high-dose steroids. Anti-3-hydroxy-3-methylglutaryl-coenzyme A (anti-HMG-CoA) reductase antibody returned strongly positive. While the patient was on steroids, her muscle weakness and CPK level gradually improved. She was discharged on oral steroids. Statin-induced autoimmune myopathy should be considered with high suspicion when there is a significantly elevated CPK level. Discontinuation of statin therapy does not lead to muscle recovery or improvement in the CPK level. Diagnosis is confirmed by positive anti-HMG-CoA reductase autoantibody and a muscle biopsy.

## Introduction

Statins are 3-hydroxy-3-methylglutaryl-coenzyme A (HMG-CoA) reductase inhibitors and lipid-lowering medications that reduce the incidence of cardiovascular disease. Statins have a well-known side effect of myopathy. Muscle pain and weakness associated with statin treatment occur in about 10 per 100,000 persons per year [1]. Rhabdomyolysis with statin monotherapy occurs only in about 3-4 per 100,000 persons per year [2]. Statin-associated autoimmune myopathy (SAAM) is a very rare condition with an incidence rate of about 2-3 per 100,000 persons per year [3]. SAAM refers to symmetrical proximal weakness and muscle pain with persistently elevated creatine phosphokinase (CPK) (>10-fold the upper limit of normal in nearly 90% of the cases). The symptoms persist even after statin therapy is stopped. HMG-CoA reductase antibody testing is used for confirming the diagnosis, and a muscle biopsy is used to determine muscle cell regeneration and necrosis [4].

## Case presentation

A 56-year-old female presented with generalized weakness. Her weakness started in the bilateral lower extremities about three weeks prior to presentation but progressively worsened. The weakness extended up to her bilateral upper extremities. She was not able to perform her activities of daily living such as feeding herself or using the bathroom. The patient also endorsed having difficulty swallowing and a headache but did not have any skin rashes, nausea or vomiting, vision changes, jaw pain or weakness in mastication, recent history of abdominal pain, diarrhea, or upper respiratory tract infection. She had a past medical history of hypertension, hyperlipidemia, cerebral aneurysm status post clipping, and seizure disorder. Her home medications included amlodipine, aspirin, atorvastatin, topiramate, and levetiracetam. The patient’s family history was unremarkable.

The patient’s vital signs on initial presentation were temperature of 36.9°C, heart rate of 73 beats per minute, respiratory rate of 18 breaths per minute, blood pressure of 127/75 mmHg, and SpO_2_ of 91% on room air. Lungs were clear to auscultation bilaterally. She had normal S1, S2, with regular rate and rhythm. The abdomen was soft and non- tender. On neuromuscular examination, she had intact cranial nerves and sensation. Her reflexes were normal. She had bilateral lower extremity strength of 1/5 and upper extremity strength of 3/5. The weakness was significant in the proximal muscles of her extremities, including the quadriceps and rotator cuff muscles. Initially, given her ascending muscle weakness and decreased SpO_2_, she underwent lumbar puncture to evaluate for Guillain-Barre syndrome before CPK level was reported. The cerebrospinal fluid chemistry and hematology study findings were normal. The patient also had some lumbar spine tenderness; however, computed tomography of the lumbar spine did not show any acute findings.

The patient was found to have elevated CPK level at 17,144 U/L. She also had severe transaminitis with alanine aminotransferase of 647 IU/L and aspartate transaminase of 599 IU/L. She had normal alkaline phosphatase and bilirubin. Given significant muscle weakness, she underwent further workup to evaluate for rheumatological disorders. The patient’s generalized weakness was thought to be secondary to a myositis such as dermatomyositis or statin-induced myopathy/necrotizing myositis. Patient had been on atorvastatin 40 mg daily for three years, which was discontinued. She was started on high-dose steroid therapy along with intravenous fluids. The patient underwent muscle biopsy, and the pathology result was consistent with necrotizing myopathy. Her rheumatologic workup was significant for elevated HMG-CoA reductase antibody at 458 units. She had negative antinuclear antibody, anti-Jo, and anti-mi-2. Anti-signal recognition antibody which is specific for necrotizing myositis was negative. A diagnosis of statin-induced autoimmune myopathy was thus confirmed.

She was administered methylprednisolone 125 mg every six hours for four days and then methylprednisolone 60 mg for one day. The patient’s symptoms and CPK levels improved, and she was discharged on prednisone 60 mg daily for two weeks. She was given outpatient rheumatology follow-up in two weeks for tapering of prednisone and evaluation for disease-modifying anti-rheumatic drugs therapy.

## Discussion

Statins are used to reduce cardiovascular risk and have mortality benefits. They are one of the most widely used medications. They inhibit the enzyme 3-hydroxy-3-methylglutaryl-CoA reductase (HMGCR). Myopathy is one of the well-known side effects that usually improves after statin discontinuation [5]. If a patient’s symptoms of pain and/or weakness are severe or a patient has marked hyperCKemia (elevation >10 times the baseline), further immediate investigation is indicated for possible SAAM [4]. Our patient had a CPK that was 85 times more than upper limit of normal.

SAAM is a rare side effect of statin therapy. The pathophysiology is not clear but is likely related to statin-induced autoimmunity against HMG-CoA reductase in genetically susceptible individuals. Patients usually present with symmetrical proximal muscle weakness and pain along with difficulty in climbing stairs or rising from a chair [3,6]. However, patients may have a varied presentation. A case of statin-induced autoimmune myopathy was reported with oropharyngeal dysphagia [7]. In another patient, statin use was associated with bilateral foot drop [4]. Although our patient had generalized weakness on presentation, she reported an ascending weakness that is not typical [8] and could also occur in Guillain-Barre syndrome. Her reflexes were normal. Lumbar puncture results were also normal [9].

The onset of SAAM ranges from weeks to years after starting statin therapy [10]. Our patient was on statin therapy for at least three years without any side effects. She had not started any new medications recently. Other risk factors for myopathy include recent strenuous activity, vitamin D deficiency, hypothyroidism, combination of statins plus fibrates, and reduced renal or hepatic function [4]. Our patient had normal thyroid stimulating hormone levels. She was only on monotherapy with atorvastatin 40 mg as an anti-lipid agent. She did not have any kidney disease. She had normal hepatic function until her presentation, and her elevated liver function tests were likely due to rhabdomyolysis and myopathy.

**Figure 1:**
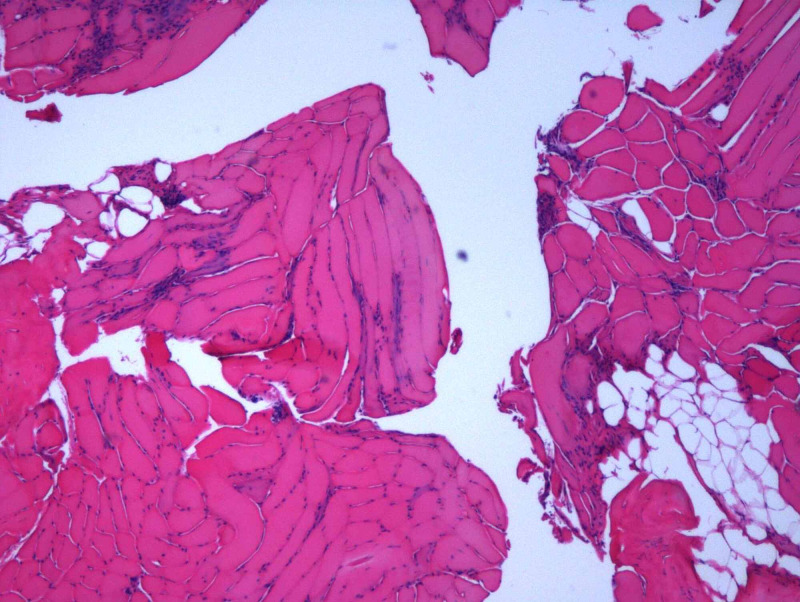
Muscle biopsy.

The diagnosis of SAAM requires high suspicion according to symptoms, examination, and labs. Anti-HMGCR antibodies are highly specific for those with an autoimmune myopathy and are not seen in the vast majority of statin-induced myopathy. The sensitivity and specificity of the anti-HMGCR enzyme-linked immunosorbent assay are 94.4% and 99.3%, respectively [11]. Anti-signal recognition particle (anti-SRP) is also specific, but is more prognostic for necrosis, severe muscle weakness, atrophy, and more frequent extramuscular symptoms [12]. Our patient had elevated anti-HMGCR at 458 units (reference range: 0-20 units) but anti-SRP was negative. Muscle edema may be present on magnetic resonance imaging (MRI) [3]. Our patient was unable to have an MRI due to her aneurysm clipping. A muscle biopsy is recommended only in patients with persistently elevated CPK level after treatment to determine muscle damage with evidence of necrosis, regeneration, and cellular infiltrates, as well as presence of macrophages, T lymphocytes (CD3+, CD4+, and CD8+), and CD123+ plasmacytoid dendritic cells [3,4]. Our patient had muscle biopsy which was consistent with necrotizing myopathy (Figure 1). The section shows skeletal muscle in both longitudinal and cross-sectional array. Muscle fibers have marked variation in fiber size with necrotic fibers, myophagocytosis, regenerative fibers, and scattered small angulate myofibers. There is no evidence of vasculitis.

In patients with muscle pain and weakness, rhabdomyolysis with a significantly elevated CPK who are taking statin therapy, if the symptoms do not resolve with statin removal, immunosuppressive therapy is indicated depending on the positivity of anti-HMGCR antibodies [4,8]. Multiple studies suggest prednisone at a dose of 1 mg/kg of bodyweight [3]. Methotrexate is commonly used if myopathy does not improve. Also, IVIG may be needed for severe cases [6]. A retrospective study showed efficacy of triple induction with corticosteroids, IVIG, and a corticosteroid-sparing immunosuppressant. This study reported that early remission is associated with maintaining remission [13]. Long-term treatment is specific for each patient. Although some patients experience relapse, the prognosis is generally good [4,14]. Our patient was started on treatment while awaiting lab results. She was treated with high-dose methylprednisolone 125 mg intravenously every six hours for four days and then continued with prednisone. She improved with steroids and her symptoms were minimal on follow-up. As far as alternative options for cholesterol management in patients with SAAM, we can consider PCSK9 inhibitors such as Praluent (alirocumab) and Repatha (evolocumab) [15].

## Conclusions

SAAM is a rare side effect of statin therapy characterized by proximal muscle weakness and significantly elevated CPK level that does not improve with statin cessation. High suspicion is essential for diagnosis. Diagnosis is confirmed by positive anti-HMG-CoA reductase autoantibody. Treatment plan includes statin discontinuation, steroids, immunosuppressive therapy, or IVIG. Although some patients have relapses or need longer treatment, the prognosis is usually good.
